# The Velvet Complex Is Essential for Sclerotia Formation and Virulence in *Sclerotinia sclerotiorum*

**DOI:** 10.3390/jof11110786

**Published:** 2025-11-01

**Authors:** Weijie Huang, Zhuo Chen, Ning Cui, Jessica Wijaya, Yan Xu, Mingsong Wu, Yuelin Zhang, Xin Li

**Affiliations:** 1 Michael Smith Laboratories, University of British Columbia, Vancouver, BC V6T 1Z4, Canada; 2 The College of Life Sciences, Sichuan University, Chengdu 610064, China; 3 Department of Botany, University of British Columbia, Vancouver, BC V6T 1Z4, Canada

**Keywords:** fungal pathogens, *Sclerotinia scleortiorum*, sclerotia, velvet complex, SsVel1, SsLae1, forward genetic analysis, UV mutagenesis

## Abstract

*Sclerotinia sclerotiorum* is a devastating soilborne fungal pathogen that causes Sclerotinia stem rot in many economically important crops. It forms sclerotia, resilient dormant structures that can persist in soil for years. Understanding the molecular mechanism of sclerotia formation is crucial for developing effective control strategies, but only a limited number of signaling components have been uncovered in this process. Through independent forward genetic screens, we identified *SsLae1* and *SsVel1*, two core components of the conserved fungal velvet complex, as essential regulators of sclerotia formation and virulence in *S. sclerotiorum*. Disruption of either gene abolished sclerotia formation, impaired compound appressorium development, and significantly reduced virulence. Further RNA-seq analysis using the *Ssvel1* mutant revealed widespread downregulation of known developmental and virulence regulators. Collectively, these findings establish the velvet complex as a master regulator for both sclerotia development and virulence in *S. sclerotiorum*.

## 1. Introduction

*Sclerotinia sclerotiorum* is a destructive plant pathogen that infects a broad range of hosts [1,2]. As a soilborne fungus, its dormant structure sclerotia can overwinter and remain viable in soil over extended periods. Infection begins when sclerotia germinate either as mycelia or form fruiting bodies, apothecia, which release airborne ascospores. When these ascospores land on plant surface, they can germinate, form appressoria to penetrate host tissues and establish infection [2,3]. Sclerotia can be formed on or inside infected tissues, repeating the disease cycle. The durability of sclerotia enables *S. sclerotiorum* to withstand adverse environments such as low temperatures until favorable conditions return. Although sclerotia are key to the lifestyle of *S. sclerotiorum*, the molecular understanding of its formation is still incomplete.

Both forward and reverse genetic analyses enable researchers to investigate molecular components involved in *S. sclerotiorum* virulence and sclerotia development. Previous studies have reported knockout (KO) mutants that failed to form sclerotia and/or exhibited reduced virulence. For example, autophagy is a conserved eukaryotic process that degrades and recycles defective proteins and organelles through the lysosomal pathway to maintain protein homeostasis. Knocking out autophagy-related genes (ATGs) individually, *SsATG1*, *SsATG2*, *SsATG4*, *SsATG5*, *SsATG8*, *SsATG9*, or *SsATG26*, leads to failed sclerotia formation and reduced virulence, particularly with *SsATG1, SsATG8*, and *SsATG26* [4,5]. In the cyclic adenosine monophosphate (cAMP) signaling pathway, cAMP levels are maintained in homeostasis by phosphodiesterase (PDE) enzymes. Knocking out the high-affinity phosphodiesterase gene *SsPDE2* not only leads to failed sclerotia formation and reduced virulence but also elevated oxalic acid (OA) secretion and excessive cAMP accumulation [6]. Downstream of cAMP, the protein kinase A (PKA) pathway controls the autophagy activities [7]. OA is a key virulence factor in *S. sclerotiorum*. Knocking out the OA biosynthesis enzyme oxaloacetate acetylhydrolase (OAH1) led to largely reduced virulence and abnormal appressoria and sclerotia development [8,9].

Similarly, the Mitogen-activated protein kinase (MAPK) cascade plays essential roles in sclerotia formation and virulence. Mutants of *SsSte50* encoding a MAPKKK adaptor, *SsSte11* encoding a MAPKKK, *SsSte7* encoding a MAPKK, and *SsSmk1* encoding a MAPK failed to form sclerotia and exhibited reduced virulence, while knocking out one of the MAPK downstream transcription factors *SsSte12* led to normal sclerotia formation but defective virulence [10]. In parallel, reactive oxygen species (ROS) play key roles in Sclerotinia biology. Mutants knocking out ROS biogenesis enzyme NADPH oxidase (SsNox1) fail to form sclerotia and show significantly reduced virulence with reduced OA production, while *SsNox2* KO mutants produce irregular sclerotia but retain normal virulence and OA concentrations [11]. In addition, RAS cycle proteins regulate cell growth, sclerotia development, and stress responses. Mutants of SsGAP1, a RAS-GTPase activating protein that inactivates SsRAS, fail to form sclerotia and exhibit significantly reduced virulence and lower OA production [12]. Furthermore, mutants of the transcription factor SsPac1 generate aberrant sclerotia, with lower OA levels and defective virulence [13].

To search for new regulators of sclerotia formation, we established a forward genetic pipeline to isolate non-sclerotia mutants and identify their causal genes. Thirty-two mutants were identified with diverse morphological defects [14]. More recently, we screened for OA-independent mutants using *Ssoah1* as a screening background [15]. Here, we report on the characterization of mutants identified from both screens with mutations in genes encoding the velvet complex components. Our data reveal the critical roles of *SsVel1* and *SsLae1* in regulating sclerotia formation, virulence, and other biological processes of *S. sclerotiorum*.

## 2. Results

### 2.1. Identification and Characterization of Three Sclerotinia sclerotiorum UV Mutants That Display Thick Aerial Hyphae, No Sclerotia, and Decreased Virulence

**Figure 1:**
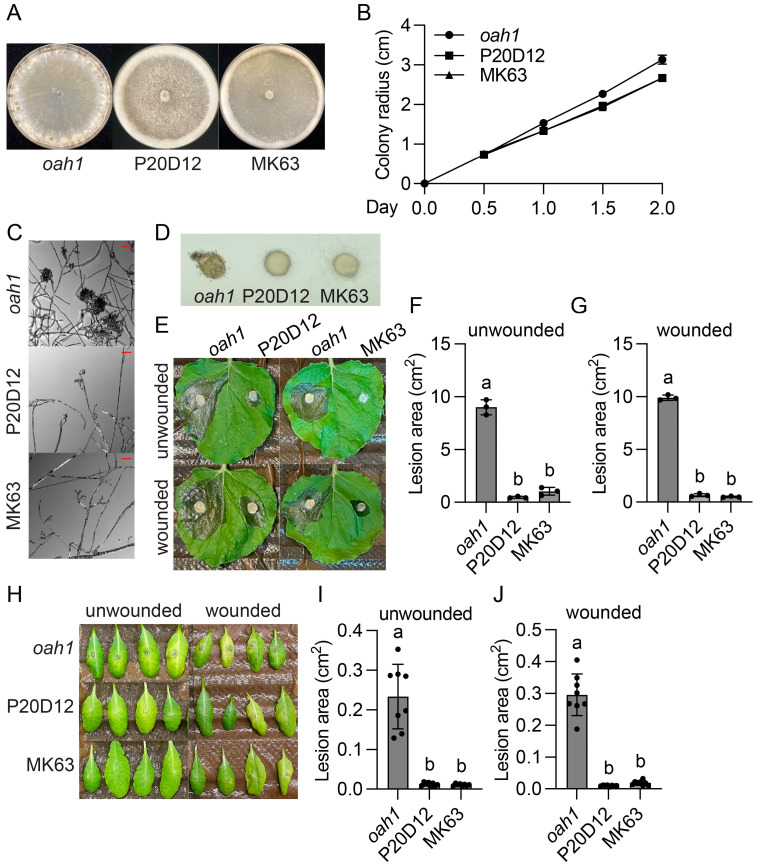
Identification and characterization of the P20D12 and MK63 mutants from UBC. (**A**) Colony morphology of the indicated genotypes. Images were captured 10 days post inoculation (dpi). (**B**) Colony radius of the indicated genotypes through time on PDA plates. (**C**,**D**) Compound appressoria of the indicated genotypes formed on glass slides (**C**) and parafilm (**D**). Images were captured at 36 hpi. The scale bar in (**C**) is 50 µm. (**E**–**G**) Pathogenicity assays of the indicated genotypes on *Nicotiana benthamiana* (**E**) and their quantitative evaluation. Images were taken at 36 hpi. Error bars represent standard deviations. Letters indicate statistical differences (*p* < 0.0001, one-way ANOVA followed by Tukey’s multiple comparison test). (**H**–**J**) Pathogenicity assays of the indicated genotypes on *Arabidopsis thaliana* (**H**) and their quantitative evaluation. Images were taken at 36 hpi. Error bars represent standard deviations. Letters indicate statistical differences (*p* < 0.0001, one-way ANOVA followed by Tukey’s multiple comparison test).

**Figure 2:**
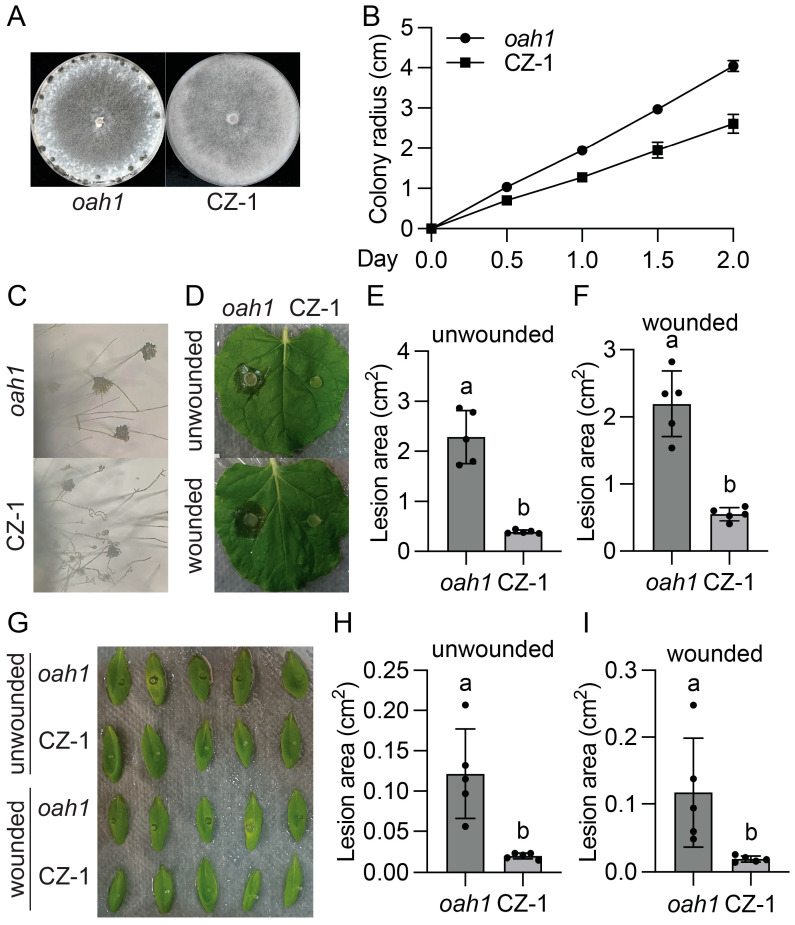
Identification and characterization of the CZ-1 mutant from SCU. (**A**) Colony morphology of the indicated genotypes. Images were captured at 10 dpi. (**B**) Colony radius of the indicated genotypes on PDA plates. (**C**) Compound appressoria of the indicated genotypes formed on glass slides. Images were captured at 36 hpi. (**D**) Pathogenicity assays of the indicated genotypes on *Nicotiana benthamiana* leaves. Images were taken at 36 dpi. (**E**,**F**) Quantitative evaluation of lesion area in (**D**). Error bars represent standard deviations. Letters indicate statistical differences (*p* < 0.0001, unpaired *t*-test). (**G**) Pathogenicity assays of the indicated genotypes on *A. thaliana* leaves. Images were taken at 36 dpi. (**H**,**I**) Quantitative evaluation of lesion area in (**G**). Error bars represent standard deviations. Letters indicate statistical differences (*p* < 0.0001, unpaired *t*-test).

To identify oxalic acid (OA) independent virulence factors, we performed two independent UV mutagenesis-based forward genetic screens using the *S. sclerotiorum oah1* mutant [15]. As shown in Figure 1A and Figure 2A, mutant P20D12 and MK63, identified at the University of British Columbia (UBC) in Canada, and CZ-1, found at Sichuan University (SCU) in China, could not produce sclerotia. Instead of growing flat mycelia as wild type (WT) or *oah1*, they grew profuse aerial hyphae, forming velvety rings around the plate edge. In addition, they grew slightly slower than the *oah1* mutant (Figure 1B and Figure 2B) and developed fewer, smaller, and deformed infection cushions (Figure 1C,D and Figure 2C). Consistent with impaired appressoria, both mutants caused smaller necrotic lesions compared with the *oah1* control when inoculated on either unwounded or wounded *Nicotiana benthamiana* leaves (Figure 1E–G and Figure 2D–F) and *Arabidopsis thaliana* leaves (Figure 1H–J and Figure 2G–I). Altogether, these data reveal that P20D12, MK63, and CZ-1 are defective in sclerotia formation, compound appressoria development, and both pre- and post-penetration virulence.

**Figure 3:**
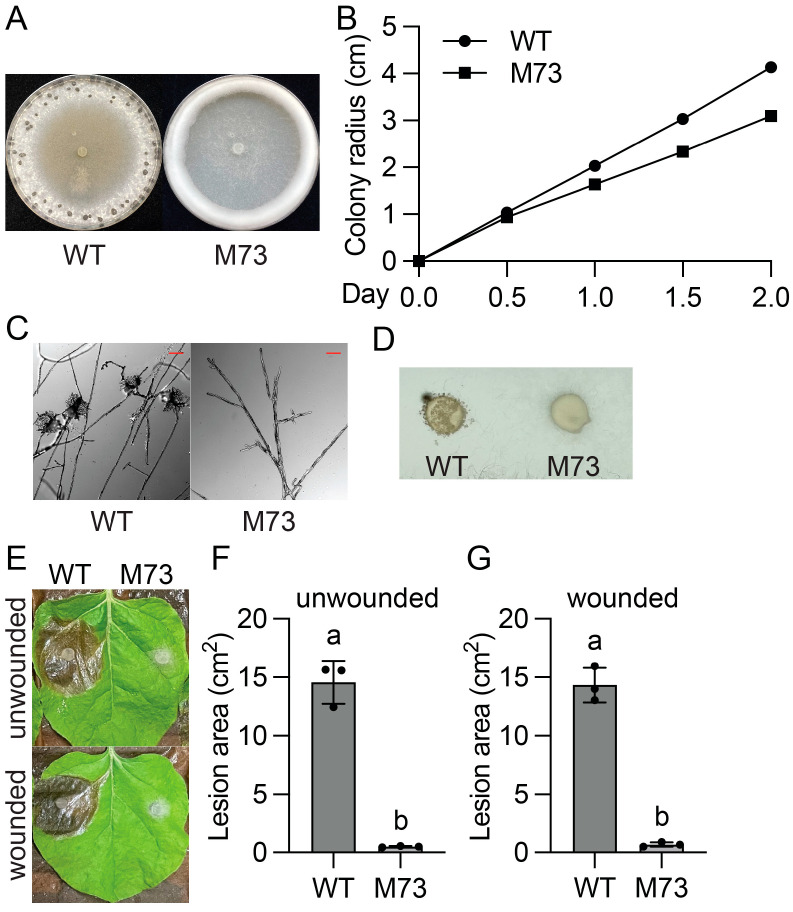
Identification and characterization of the M73 mutant. (**A**) Colony morphology of the indicated genotypes. Images were captured at 10 dpi. (**B**) Colony radius of the indicated genotypes on PDA plates. (**C**,**D**) Compound appressoria of the indicated genotypes formed on glass slides (**C**) and parafilm (**D**). Images were captured at 36 hpi. The scale bar in (**C**) is 50 µm. (**E**) Pathogenicity assays of the indicated genotypes on *Nicotiana benthamiana* leaves. Images were taken at 36 dpi. (**F**,**G**) Quantitative evaluation of lesion area in (**E**). Error bars represent standard deviations. Letters indicate statistical differences (*p* < 0.0001, unpaired *t*-test).

M73, a mutant isolated from our earlier screen using the WT *S. sclerotiorum* 1980 strain [14], phenocopied P20D12 and MK63. It exhibited a characteristic ring of aerial hyphae (Figure 3A), produced defective appressoria (Figure 3C,D), and showed markedly reduced virulence (Figure 3E–G).

### 2.2. Molecular Cloning of SsLae1

**Figure 4:**
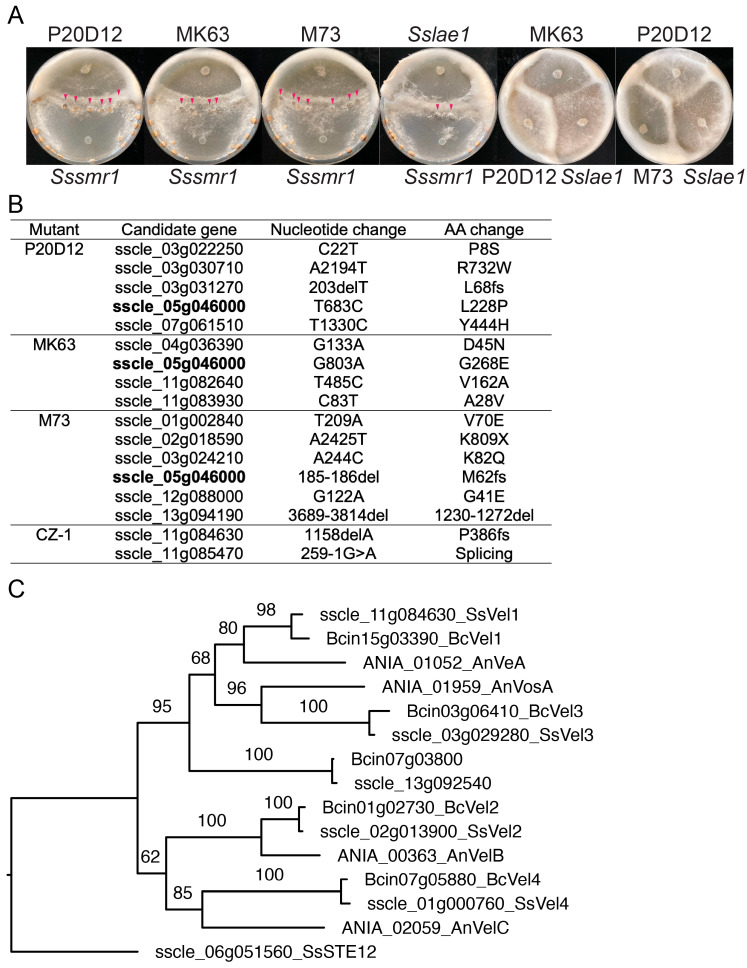
Mutations in *SsLae1* and *SsVel1* were identified in four UV mutants. (**A**) Allelism test of P20D12, MK63, M73, and *Sslae1-4* by hyphal fusion. *Sssmr1* was used as a positive control. Images were taken at 14 dpi. Red arrowheads highlighted black sclerotia formed after successful complementation. (**B**) List of mutations in candidate genes from NGS results of four UV mutants. The commonly mutated gene is bolded. (**C**) Phylogenetic tree of SsVel1 orthologues in *A. nidulans*, *B. botrytis*, and *S. sclerotiorum*. Full-length protein sequences were aligned using MUSCLE with default settings. Maximum likelihood tree was built using RAxML by means of JTT substitution model with 1000 bootstraps. SsSTE12 was used as an outgroup.

The similar phenotypes observed in mutants P20D12, MK63, and M73 prompted us to perform an allelism test on them. As shown in Figure 4A, these three mutants failed to form sclerotia between each other. As a control, black sclerotia were observed when the mycelia of these three mutants were fused with that of the *Sssmr1* mutant, which forms pink sclerotia. These results suggest that mutants P20D12, MK63, and M73 are allelic to each other, each likely carrying different mutations in the same gene.

To identify the causal mutations in P20D12, MK63, and M73, next-generation sequencing was performed using their genomic DNA. Among the candidate mutations, *Sscle_05g046000* stood out, as it was mutated in all three mutants (Figure 4B). Two-point mutations leading to Leu228Pro and Gly268Glu changes were found in P20D12 and MK63, respectively, while a deletion causing a frame shift was identified in M73.

*sscle_05g046000* encodes a protein from the family of S-adenosyl-L-methionine-dependent methyltransferases. Protein sequence alignment showed that it is an orthologue to *Lae1* of *Botrytis cinerea* and *Aspergillus nidulans* (Supplemental Figure S1). Therefore, the P20D12, MK63, M73, and two later independent KO mutants were named hereafter as *Sslae1-1*, *-2*, *-3*, *-4*, and *-5*.

**Figure 5:**
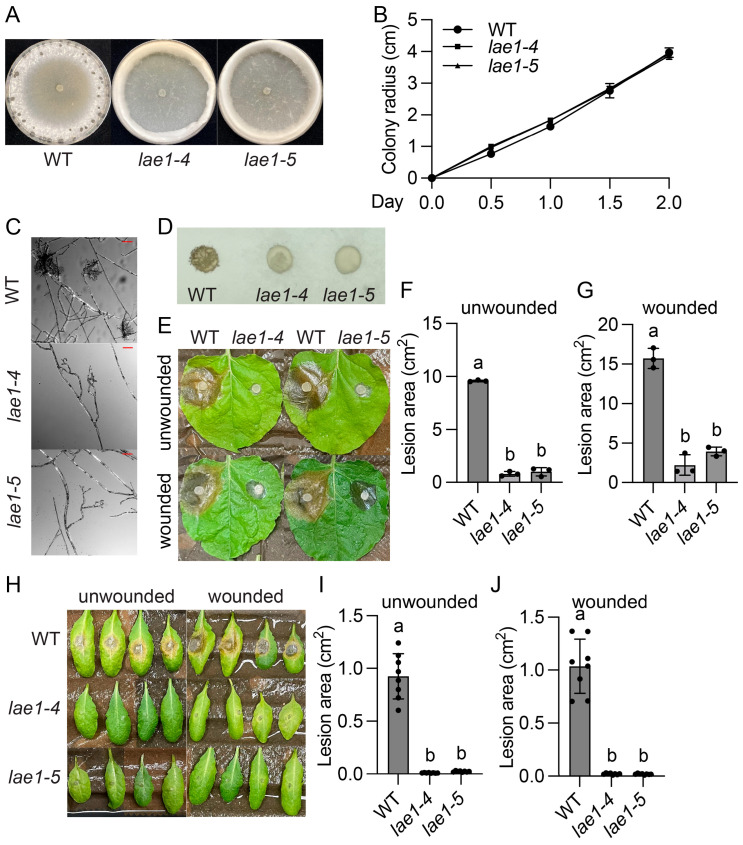
Knocking out *SsLae1* disrupted sclerotia formation, appressorium development, and virulence. (**A**) Colony morphology of the indicated genotypes. Images were captured at 10 dpi. (**B**) Colony radius of the indicated genotypes through time on PDA plates. (**C**,**D**) Compound appressoria of the indicated genotypes formed on glass slides (**C**) and parafilm (**D**). Images were captured at 36 hpi. The scale bar in (**C**) is 50 µm. (**E**–**G**) Pathogenicity assays of the indicated genotypes on *Nicotiana benthamiana* (**E**) and their quantitative evaluation. Images were taken at 36 hpi. Error bars represent standard deviations. Letters indicate statistical differences (*p* < 0.0001, one-way ANOVA followed by Tukey’s multiple comparison test). (**H**–**J**) Pathogenicity assays of the indicated genotypes on *Arabidopsis thaliana* (**H**) and their quantitative evaluation. Images were taken at 36 hpi. Error bars represent standard deviations. Letters indicate statistical differences (*p* < 0.0001, one-way ANOVA followed by Tukey’s multiple comparison test).

To test whether *sscle_05g046000* is the causal gene, deletion knockout (KO) mutants were generated (Supplemental Figure S2). Like P20D12, MK63, and M73, mycelia of the two independent deletion alleles of *sscle_05g046000* formed aerial hyphae rings at the colony edge (Figure 5A). Their growth rates were comparable to that of WT (Figure 5B). In addition, the KO mutants exhibit defects in compound appressorial development (Figure 5C,D), leading to attenuated virulence on leaves of *N. benthamiana* and *Arabidopsis* (Figure 5E–J). Thus, knocking out *sscle_05g046000* resulted in mutant phenotypes similar to P20D12, MK63, and M73, confirming it as the causal gene for the three UV mutants.

### 2.3. SsVel1 Is Also Required for Sclerotia Formation, Compound Appressorium Development, and Virulence of S. sclerotiorum

**Figure 6:**
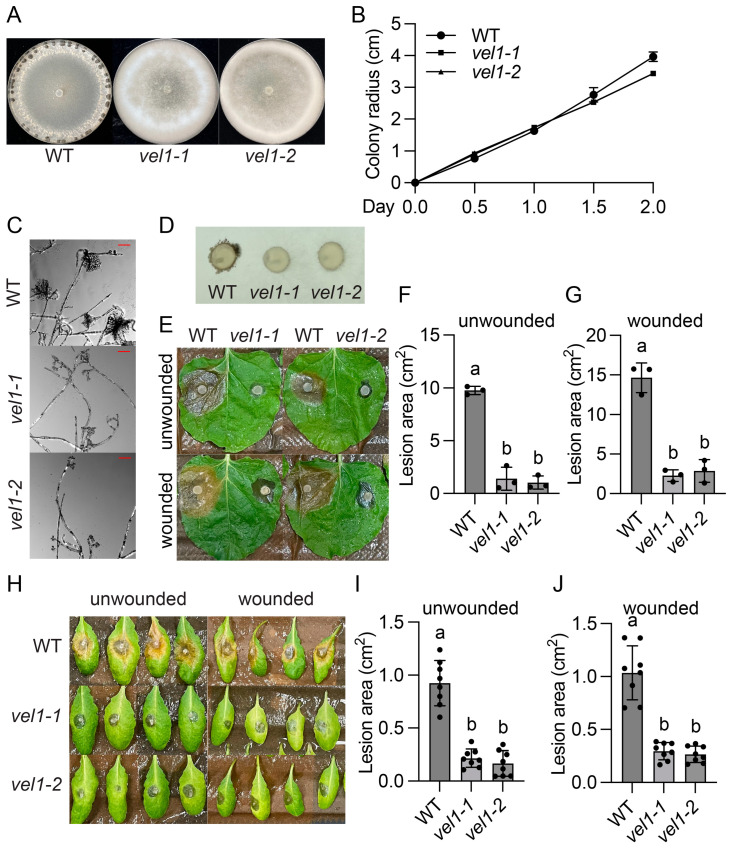
*SsVel1* mutants were defective in sclerotia formation, appressorium development, and virulence. (**A**) Colony morphology of the indicated genotypes. Images were captured at 10 dpi. (**B**) Colony radius of the indicated genotypes through time on PDA plates. (**C**,**D**) Compound appressoria of the indicated genotypes formed on glass slides (**C**) and parafilm (**D**). Images were captured at 36 hpi. The scale bar in (**C**) is 50 µm. (**E**–**G**) Pathogenicity assays of the indicated genotypes on *Nicotiana benthamiana* (**E**) and their quantitative evaluation. Images were taken at 36 hpi. Error bars represent standard deviations. Letters indicate statistical differences (*p* < 0.0001, one-way ANOVA followed by Tukey’s multiple comparison test). (**H**–**J**) Pathogenicity assays of the indicated genotypes on *Arabidopsis thaliana* (**H**) and their quantitative evaluation. Images were taken at 36 hpi. Error bars represent standard deviations. Letters indicate statistical differences (*p* < 0.0001, one-way ANOVA followed by Tukey’s multiple comparison test).

LaeA, the orthologue of SsLae1 in *Aspergillus nidulans*, was reported to physically associate with VeA and VelB proteins to form the velvet complex [16]. In the NGS data of the CZ-1 mutant, a frameshift mutation in *sscle_11g084630* was identified, which, based on our phylogenetic analysis and protein sequence alignment, corresponds to the VeA orthologue in *S. sclerotiorum* (Figure 4B,C, Supplemental Figure S3). Deletion KO mutants of *SsVel1* were obtained by homologous recombination (Supplemental Figure S2). As shown in Figure 6A, both mutants displayed similar aerial hyphal rings rather than forming sclerotia. Mycelial growth of *Ssvel1* mutants was largely similar with that of WT (Figure 6B). In addition, *Ssvel1* mutants developed fewer and deformed compound appressoria (Figure 6C,D), which can account for their impaired virulence on intact *N. benthamiana* and *Arabidopsis* leaves (Figure 6E–J). *Ssvel1* mutants caused slightly larger necrotic lesions on wounded than intact *N. benthamiana* and *Arabidopsis* leaves, but they were significantly smaller than those caused by WT (Figure 6E–J). These findings suggest that like the *Sslae1* mutants, *Ssvel1* mutants are defective in both sclerotia formation and pre- and post-penetration virulence. As LAE1 and VEL1 are part of the velvet complex in other fungi [16,17,18], the almost identical defects of the *Sslae1* and *Ssvel1* mutants suggest the two encoded proteins likely also form a velvet transcriptional complex in *S. sclerotiorum*.

### 2.4. SsVel1 Broadly Regulates Genes Specific to Infection and Sclerotia Development

**Figure 7:**
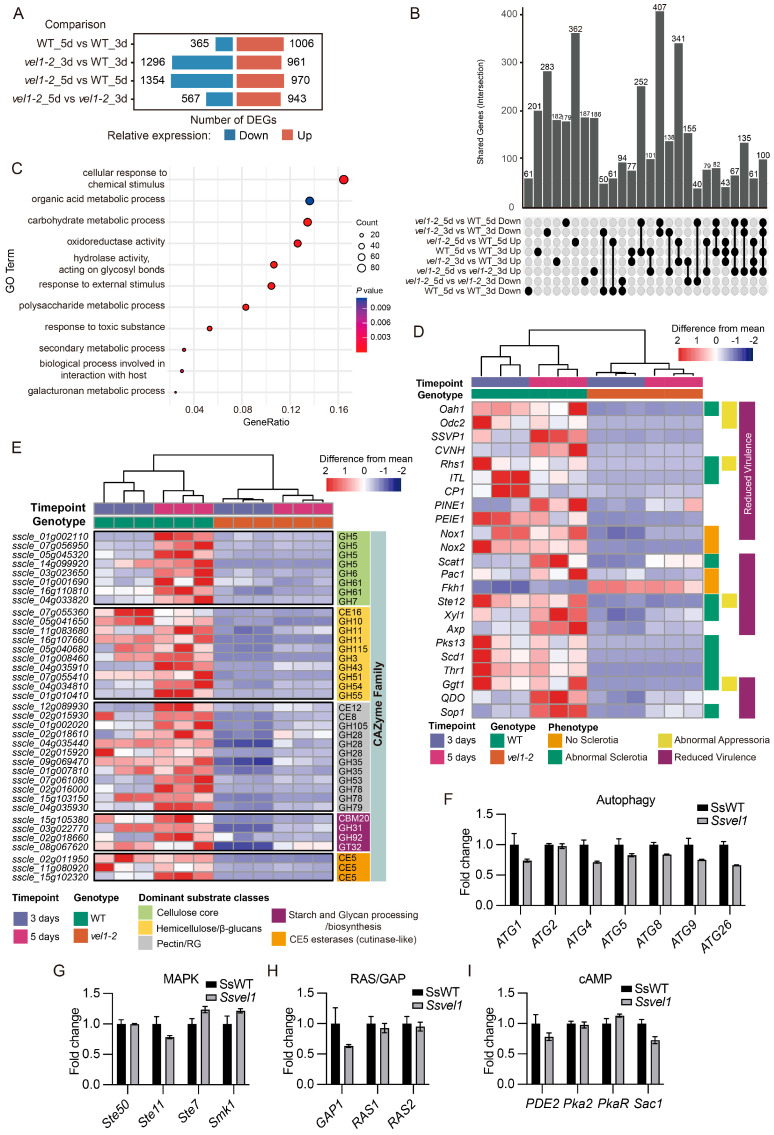
RNA-seq analysis of WT and *Ssvel1-2*. (**A**) Comparisons of significantly differentially expressed genes (DEGs) between different genotypes and ages. Numbers represent the DEG count for the indicated categories. The thresholds used were log_2_FC > 1.0 or <−1.0 and padj < 0.05. (**B**) Upset plot showing DEGs shared between different groups. Vertical bars and corresponding values represent the distinct set between sets labeled with gray dots. (**C**) Gene ontology (GO) enrichment analysis of *SsVel1*-dependent genes. Eleven significantly enriched GO terms are shown. (**D**) Expression profile comparisons of known genes involved in sclerotia formation, compound appressorium development, and virulence. *S. sclerotiorum* gene codes are listed on the left. Mutant phenotypes are denoted on the right by distinct colors. Hierarchical clustering of samples was completed and is represented by the dendrogram above the heatmap. Different genotype and age groupings are denoted by colored bars on top of the heatmap. (**E**) Expression profile comparisons of the genes encoding predicted cell wall-degrading enzymes. *S. sclerotiorum* gene codes are listed on the left. Substrate classes and CAZyme family are denoted on the right. Hierarchical clustering of samples was completed and is represented by the dendrogram above the heatmap. Different genotype and age groupings are denoted by colored bars on top of the heatmap. (**F**–**I**) Relative expression of indicated genes in different signaling pathways as indicated. The transcript levels of WT are normalized to 1. Expression data are derived from samples collected on day 5.

To gain deeper insights into how the velvet complex contributes to virulence and development in *S. sclerotiorum*, we performed RNA-seq analysis of the *vel1* mutant grown on PDA plates at both days 3 and 5 post inoculation (dpi). Among the 11,130 annotated genes in the genome of *S. sclerotiorum* strain 1980, we identified 2257 differentially expressed genes (DEGs) in the *vel1* mutant compared with the WT at 3 dpi, including 961 up-regulated and 1296 down-regulated genes (Figure 7A). At 5 dpi, the number of DEGs slightly increased to 2324, with 970 up-regulated and 1354 down-regulated (Figure 7A,B). Gene ontology (GO) enrichment analysis of these DEGs revealed significant overrepresentation of terms associated with responses to chemical and external stimuli, toxic substances, secondary metabolism, biological processes involved in interaction with hosts, and the galacturonan metabolic process (Figure 7C).

To specifically examine genes known to contribute to pathogenicity and sclerotia formation in *S. sclerotiorum*, we analyzed their expression profiles [8,10,11,19,20,21,22,23,24,25,26,27,28,29,30,31,32,33,34,35,36,37]. Notably, the expression of many established regulators was strongly dependent on *SsVel1*, including the OA biosynthesis gene *OAH1*, the transcription factors *SsSte12* and *SsPac1*, the NADPH oxidases *SsNox1* and *SsNox2*, and several known secreted effector genes (Figure 7D). These findings demonstrate that the velvet complex broadly regulates genes involved in infection and sclerotia development.

Cell wall-degrading enzymes (CWDEs) are important virulence factors in *S. sclerotiorum*, as they can degrade complex plant cell wall components, including cellulose, hemicellulose, and pectin. Many genes encoding potential CWDEs have been reported to be up-regulated during colonization of sunflower cotyledons [38]. By examining the transcripts of these CWDE genes in the *vel1-2* mutant, we found that 37 of them were down-regulated in *vel1-2* (Figure 7E), suggesting that SsVel1 widely regulates the expression of CWDE genes.

Previous studies have highlighted multiple signaling pathways implicated in sclerotia development and virulence, such as autophagy-related components, the SsSte50-SsSte11–SsSte7–SsSmk1 MAPK cascade, RAS cycle, and the cAMP-PKA pathway. Interestingly, the expression of genes in these pathways was largely unaffected in the *vel1* mutant (Figure 7F–I), suggesting that they may act upstream of, or in parallel with, the velvet complex.

## 3. Discussion

The velvet complex was first reported in *Aspergillus nidulans*, where it integrates light signals to regulate diverse processes, including sexual and asexual development, virulence, and secondary metabolism [39]. In recent years, functionally similar velvet complexes have been identified in other fungi such as *Neurospora crassa*, *Fusarium fujikuroi*, *F. oxysporum*, and *Botrytis cinerea* [16,18,40,41,42,43,44,45,46,47,48]. In this study, our forward genetic screens revealed that *SsLae1* and *SsVel1* encoded part of the velvet complex that seems to be essential for compound appressorial development, virulence, and sclerotia formation in *S. sclerotiorum*.

**Figure 8:**
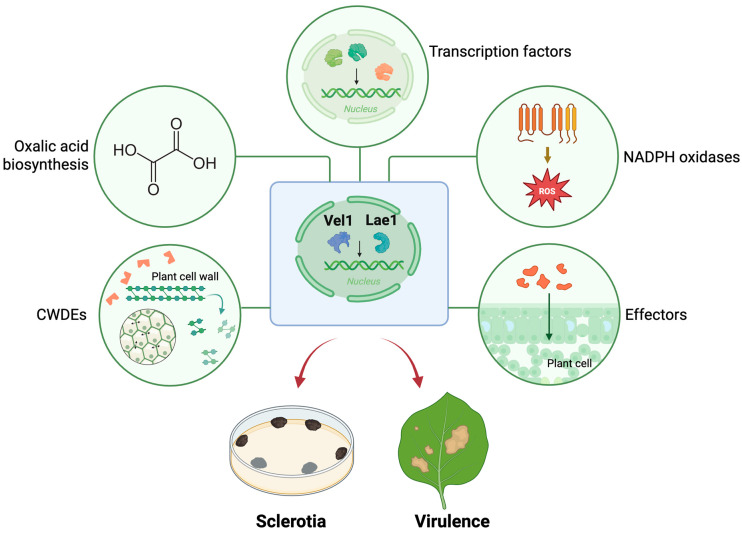
A regulatory model of SsLae1 and SsVel1. Created in BioRender. Liu, X. (2025) https://BioRender.com/qvb10wb.

Loss of *SsLae1* or *SsVel1* dramatically compromises virulence. During early colonization, *S. sclerotiorum* develops compound appressoria, the specialized infection structures that release cell wall-degrading enzymes and effectors to damage host cells, suppress immunity, and promote tissue maceration and necrosis [1,2,3]. Infection assays revealed that *SsLae1* and *SsVel1* mutants produced markedly smaller lesions on intact leaves compared with the wild type, consistent with their deformed compound appressoria. On wounded leaves, these mutants also caused restricted necrosis, whereas wild-type infections spread beyond the inoculation site and ultimately led to host wilting. This phenotype resembles that of the *Ssoah1* mutant, which is defective in OA production [8]. Indeed, RNA-seq analysis showed that *OAH1* expression failed to be up-regulated in the absence of *SsVel1*. In addition, additional known virulence-associated genes are also under the control of the velvet complex, including the plasma membrane-localized NADPH oxidases *SsNOX1/2* [11], transcription factors *SsSte12* and *SsPac1* [10,13], secreted effectors SsCVNH, SsCP1, SsPINE1, and SsPEIE1 [29,30,32,37], and other genes predicted to be responsive to hydrogen peroxide under the GO term response to toxic substance, which may help counteract host-derived reactive oxygen species. Collectively, these results establish the velvet complex as a master regulator of multiple virulence pathways in *S. sclerotiorum* (Figure 8).

Interestingly, disruption of the velvet complex completely abolished sclerotia formation in *S. sclerotiorum*. No mycelial aggregates can be observed in the *SsLae1* and *SsVel1* mutants, which instead continued vegetative growth. This demonstrates that the velvet complex is indispensable for sclerotia initiation. RNA-seq analysis revealed that several known regulators of sclerotia formation, including the NADPH oxidases *SsNox1* and *SsNox2* [11] and the transcription factors *SsFkh1* and *SsPac1* [13,36], were downregulated in *Ssvel1* knockout mutants, providing mechanistic insights into how the velvet complex governs this developmental process (Figure 8).

Redundancy within the velvet complex has been reported in several fungal species. In *A. nidulans*, both VelB and VelC are components of the complex [16,41]. In *Botrytis cinerea*, three VeA orthologues have been identified—*BcVel1*, *BcVel2*, and *BcVel3*. Disruption of either *BcVel1* or *BcVel2* leads to reduced pathogenicity and decreased organic acid production, whereas the deletion of *BcVel3* shows little effect [44]. Based on amino acid sequence similarity, homologues of *SsVel1* besides *SsLae1* are present in *S. sclerotiorum* (Figure 4C and Supplemental Figure S3). Whether they contribute to the velvet complex and share similar functions remains unclear. Nevertheless, the severe developmental defects observed in the absence of *SsVel1* and *SsLae1* suggest that they may be the predominant members of the velvet complex in *S. sclerotiorum*.

Although the velvet complex has been studied for more than a decade, many aspects of its regulation and function remain poorly understood. For example, the upstream components and mechanisms that activate the velvet complex have not yet been identified. *Lae1* and *Vel1* are believed to function as a methyltransferase and a transcription factor, respectively, and can physically associate with one another [39]. However, their precise molecular roles in vivo remain unclear. Previous studies have also suggested that *Lae1* and *Vel1* exhibit both overlapping and distinct functions, as their mutant phenotypes are not always identical. Future analyses, such as ChIP-seq and whole-genome bisulfite sequencing, will be instrumental in elucidating how these proteins coordinate transcriptional regulation, and whether they influence DNA methylation. It is notable that the velvet complex is known to mediate light responses in several fungi. However, based on our observations and a previous report [28], sclerotia development and pathogenicity of *S. sclerotiorum* do not appear to be light-dependent. This raises the possibility that the *S. sclerotiorum* velvet complex is activated through mechanisms distinct from light. Future examinations are needed to elaborate how the velvet complex is activated for sclerotia formation and virulence in *S. sclerotiorum*.

Given the central role of the velvet complex in both virulence and sclerotia development, the RNA-seq datasets generated in this study provide a valuable resource for identifying novel regulators associated with fungal development, pathogenicity and survival. Moreover, the velvet complex itself represents a promising target for disease management. Recent studies have provided evidence that the host-induced gene silencing (HIGS) of several virulence-related genes effectively reduces lesion size on *N. benthamiana* leaves infected by *S. sclerotiorum* [6,10,12]. Targeting *SsLae1* and *SsVel1* through similar approaches may offer a viable strategy to control Sclerotinia stem rot in crops. Future studies will be needed to evaluate the feasibility and effectiveness of such approaches in planta.

## 4. Materials and Methods

### 4.1. Fungal Strains and Culture Conditions

The WT strain S. *sclerotiorum* 1980 and *oah1* strain were generously shared by Dr. Jeffery Rollins from the University of Florida [31]. All mutants with different backgrounds and WTs were grown on potato dextrose agar (PDA, potato dextrose broth, Shanghai Bio-Way Technology, Shanghai, China, and Agar A, BIO BASIC, Markham, ON, Canada) at room temperature and were stored on PDA slants at 4 °C or as sclerotia. For transformant screening, hygromycin B (MilliporeSigma, Oakville, ON, Canada) was added to a final concentration of 50 µg/mL.

### 4.2. UV Mutagenesis

UV mutagenesis was performed following the method described previously [14]. In short, ascospores of the *S. sclerotiorum* strain 1980 WT and *Ssoah1* were obtained from apothecia [49], resuspended in sterilized potato dextrose broth (PDB) (Shanghai Bio-Way Technology, Shanghai, China) and diluted to 10^4^ spores/mL after counting with a hemocytometer. Spore suspensions (300 µL) were spread onto PDA plates (SARSTEDT Petri dishes, 92 × 16 mm, Cat. No. 82.1473.001, SARSTEDT, Nümbrecht, Germany), dried, and irradiated with UV light (TL-2000, 9000 mJ/cm^2^, 15 s, Taylor Scientific, St. Louis, MO, USA). Plates were incubated at room temperature for 2 days, after which individual colonies were transferred to 96-well plates containing PDA. Mutants without sclerotia were identified after about 7 days, and transferred onto fresh PDA, and incubated for 2 weeks to confirm morphological defects.

### 4.3. Genomic DNA Extraction and NGS Analysis

Mycelia of *S. sclerotiorum* mutants were cultured in PDB for 2–3 days, harvested, and ground into fine powder in liquid nitrogen. Genomic DNA was extracted using the CTAB method [14]. DNA quality was assessed on 1% agarose gels. Library construction, sequencing, and read quality control were performed by Novogene (Beijing, China) using the Illumina NovaSeq 6000 platform (San Diego, CA, USA), generating 14–17 million clean paired-end reads per sample. Clean reads were aligned to the *S. sclerotiorum* reference genome (ASM185786v1) with BWA-MEM [50,51]. Variants were identified using SAMtools with default parameters [52] and annotated according to GATK best practices for germline short variant discovery (SNPs and indels) [53].

### 4.4. Target Gene Knockout

*SsLae1* and *Ssvel1* knockouts were generated by homologous recombination. In brief, ~1500 bp upstream and downstream fragments of the target gene were amplified with WT genomic DNA and fused with the *hph* fragments amplified from the *pCH-EF-1* plasmid (provided by Dr. Daohong Jiang, Huazhong Agricultural University, China) to generate the upstream-*hph*-downstream knockout cassette [54]. All primers used in this study are listed in Supplemental Table S1. The knockout cassette was then transformed into WT protoplasts. Transformants were obtained on PDA plates supplemented with hygromycin B at a final concentration of 50 µg/mL, and individual colonies were transferred to fresh selective plates. Colony PCR was performed to confirm *hph* insertion. To obtain homozygous mutants, selected transformants were cultured in PDB overnight, and fresh mycelia were used to generate protoplasts. The resulting protoplasts were diluted and spread on PDA plates with hygromycin B. Germinating colonies were transferred individually to selective plates for PCR confirmation. Pure knockout transformants were retained for subsequent experiments.

### 4.5. Colony Morphology and Growth Rate Determination

The WT strain, UV-induced mutants, and knockout mutants were cultured on PDA plates for 2–3 days. Mycelial agar plugs (5 mm diameter) were taken from the colony margin using a sterile hole punch and placed at the center of fresh PDA plates (92 mm diameter). Plates were incubated at room temperature, and colony diameters were recorded per 12 h until the mycelia reached the plate edge. Colony morphology was documented at 14 days post inoculation (dpi) except as indicated.

### 4.6. Plant Infection Assay

Pathogenicity was assessed on detached leaves of 4-week-old *Nicotiana benthamiana* and *Arabidopsis thaliana* (ecotype Col-0). Mycelia agar plugs (5 mm diameter for *N. benthamiana* and 2 mm diameter for *A. thaliana*) were inoculated on detached leaves arranged on moistened paper towels in a tray sealed with plastic wraps. Disease symptoms, represented by lesion area, were recorded at 24–36 hpi. Each assay was repeated two times with consistent results.

### 4.7. Compound Appressoria Formation

Compound appressoria were induced by arranging 5 mm agar plugs with actively extending hyphae onto glass slides or parafilm, followed by incubation in a high-humidity chamber for 24 h. Compound appressoria formed on glass slides were subsequently observed and photographed with a ZEISS AXIO Imager M2 microscope (Jena, Germany) with a 63× objective lens. Compound appressoria formed on parafilm were photographed using a smartphone digital camera.

### 4.8. RNA-seq Analysis

Wild-type and *Ssvel1* strains were first cultured on PDA plates (92 mm diameter) for 2 days at room temperature in the dark. Actively growing mycelia were then transferred onto fresh PDA plates with cellophane and incubated further for 3 and 5 days, respectively, under the same conditions. Each condition was sampled in three biological replicates. Total RNA was isolated using TRIzol reagent (Invitrogen, Carlsbad, CA, USA), and RNA quality was evaluated by 1% agarose gel electrophoresis and with the RNA Nano 6000 kit on the Agilent Bioanalyzer 2100 system (Agilent Technologies, Santa Clara, CA, USA).

RNA-seq library construction, sequencing, and quality assessment were carried out by Novogene using the Illumina platforms, yielding 20–23 million paired-end clean reads per sample. Reads were aligned to the *S. sclerotiorum* reference genome (ASM185786v1) using HISAT2 v2.2.0 with default parameters [55]. BAM files were processed with SAMtools v1.10 [52], and transcript counts were obtained with featureCounts from the Subread v2.0.1 package [56]. GO term enrichment was analyzed with Fisher’s exact test using annotations generated by Blast2GO [57]. Differentially expressed genes (DEGs) were identified with DESeq2 v1.28.1 [58]. Gene expression patterns were visualized using R packages, including ComplexHeatmap, UpSetR, and ggplot2 [59,60,61].

The list of differentially expressed genes is attached in Supplemental Table S2.

### 4.9. Statistical Analysis

Error bars in all figures represent standard deviations. The number of biological replicates is indicated in the figure legends. Statistical comparisons among different samples were performed either by one-way ANOVA with Tukey’s honestly significant difference post hoc test or by Student’s *t*-test, as reported in the figure legends.

## Data Availability

The original contributions presented in this study are included in the article/supplementary material. Further inquiries can be directed to the corresponding author.

## Supplementary Materials

The following supporting information can be downloaded at https://www.mdpi.com/article/10.3390/jof11110786/s1: Figure S1: Protein sequence alignment of SsLae1 orthologues. Full length amino acid sequences are aligned by Clustal Omega. Alignment is presented by ESPript 3.0; Figure S2: PCR verification of the homozygosity of the *SsLae1* (A) and *SsVel1* (B) knock-out mutants. Primers 5F, 6R, 7F, and 8R are specific to the *SsLae1* or *SsVel1* genes, as indicated. H855-nF and H855-nR are primers specific to the hygromycin B resistance gene *hph*. Primer sets 1 and 2 were used to verify the insertion of the *hph* cassette into *SsLae1* or *SsVel1*, respectively. Primer set 3 were used to test the homozygosity of the *SsLae1* or *SsVel1* knock-out mutants, respectively. Wild type (WT) genomic DNA was used as a control. Primer sequences are listed in Supplemental Table S1. M, DNA marker; Figure S3: Protein sequence alignment of SsVel1 orthologues. Consensus sequences are displayed. Full length amino acid sequences are aligned by Clustal Omega. Alignment is presented by ESPript 3.0; Table S1: List of primers used in this study; Table S2: (a) Differential expression analysis between *Ssvel1-2*_5d vs *Ssvel1-2*_3d performed with DESeq2 for genes in *S. sclerotiorum* genome (ASM185786v1); (b) Differential expression analysis between SsWT_5d vs SsWT_3d performed with DESeq2 for genes in *S. sclerotiorum* genome (ASM185786v1); (c) Differential expression analysis between *Ssvel1-2*_3d vs SsWT_3d performed with DESeq2 for genes in *S. sclerotiorum* genome (ASM185786v1); (d) Differential expression analysis between *Ssvel1-2*_5d vs SsWT_5d performed with DESeq2 for genes in *S. sclerotiorum* genome (ASM185786v1).
