# Supplementary material for: The Velvet Complex Is Essential for Sclerotia Formation and Virulence in Sclerotinia sclerotiorum
Source: J Fungi (Basel). 2025 Nov 1;11(11):786. doi: 10.3390/jof11110786 (PMC12653243; doi:10.3390/jof11110786)
Supplement: Supplementary file 1 [file jof-11-00786-s001.zip › jof-3946736-supplementary.pdf]

Supplemental Figures

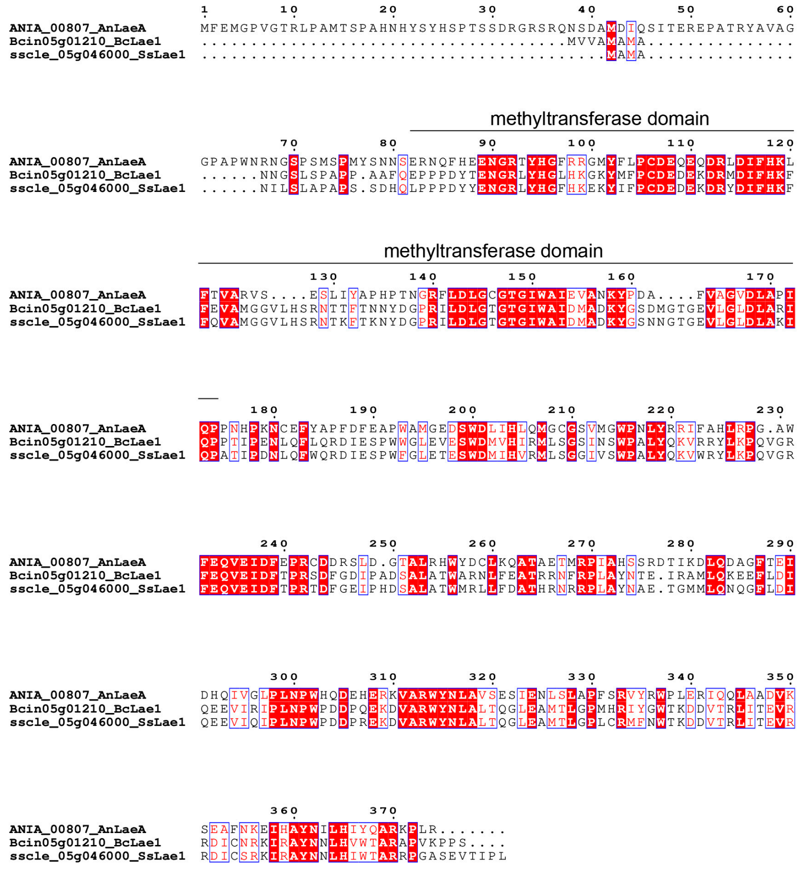

Supplemental Figure S1. Protein sequence alignment of SsLae1 orthologues. Full length amino acid sequences are aligned by Clustal Omega. Alignment is presented by ESPript 3.0.

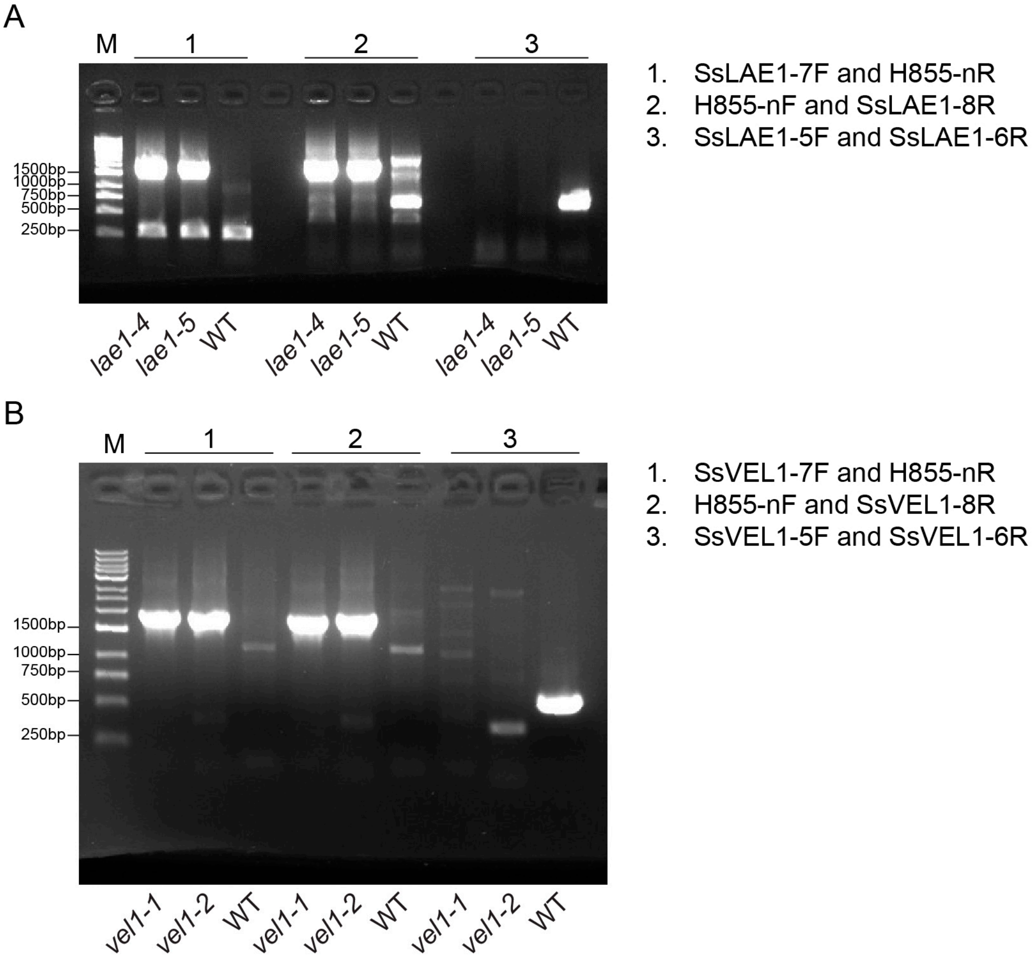

Supplemental Figure S2. PCR verification of the homozygosity of the *SsLae1* (A) and *SsVel1* (B) knock-out mutants. Primers 5F, 6R, 7F, and 8R are specific to the *SsLae1* or *SsVel1* genes, as indicated. H855-nF and H855-nR are primers specific to the hygromycin B resistance gene *hph*. Primer sets 1 and 2 were used to verify the insertion of the *hph* cassette into *SsLae1* or *SsVel1*, respectively. Primer set 3 were used to test the homozygosity of the *SsLae1* or *SsVel1* knock-out mutants, respectively. Wild type (WT) genomic DNA was used as a control. Primer sequence are listed in Supplemental Table S1. M, DNA marker.

```
                                                                                   1         10
ANIA_01052_AnVeA          ..................................................MQQPKRARAC
Bcin15g03390_BcVel1       ....................MAASIGPKSIPETITRQTKGGRKLKYTLTVIQQPERARAC
sscle_11g084630_SsVel1    MAERNITIASNHFRGSVDSNMAASTSPKSIPEIITRTTKGGRKLRYTLTVIQQPERARAC
consensus>70              ...................................................QQP.RARAC

                                    20        30        40        50        60        70
ANIA_01052_AnVeA          GQGSKSHTDRRPVDPPPVIELNIFESDPHDDSNKTDITFVYNANFFLFATLEPERPIATG
Bcin15g03390_BcVel1       GSGAKSSADRRPVDPPPVVQLRIYDET..DPRQEKEITFHYNANFFLFATLEVARNIAQG
sscle_11g084630_SsVel1    GSGAKSSADRRPVDPPPVVQLRIHDET..EPRQEKEITFHYNANFFLFATLEVARPIAQG
consensus>70              G.G.KS..DRRPVDPPPV!#L.I.#....#..#..#ITF.YNANFFLFATLE..R.IA.G

                                                                        Velvet domain
                                                                  ______________________
                                      80        90        100       110       120
ANIA_01052_AnVeA          KLMTN.QGSPVLTGVPVAGVAYLDKPNRAGYFIFPDLSVRNEGSYRFSFHLFEQIKDPKD
Bcin15g03390_BcVel1       RVQTSAPQAPVLTGMPVSGMAYLDRPNEAGYFIFPDLSVRHEGQYKLSFNLYEETKEEKD
sscle_11g084630_SsVel1    RVQTSAPQAPVLTGMPVSGMAYLDRPNEAGYFIFPDLSVRHEGQYKLSFNLYEETKEEKD
consensus>70              ...T.....PVLTG.PV.G.AYLD.PN.AGYFIFPDLSVR.EG.Y..SF.L%E#.K#.KD

                                              Velvet domain
                          ____________________________________________________________
                        130        140       150       160       170       180
ANIA_01052_AnVeA          ATEGTQPM..PSPVPGKLSSPQEFLEFRLEVISNPFIVYSAKKFPGLTTSTPISRMIAEQ
Bcin15g03390_BcVel1       TDIEPSNDSSMRQMSSAAAAAESSFDWRMELKSDQFTVYSAKKFPGLSESTDLSRTVAEQ
sscle_11g084630_SsVel1    VDIEPSNDSSMRKMSSAAAAAESSFDWRMELKSDQFTVYSAKKFPGLSESTDLSRTVAEQ
consensus>70              ......................#...#.R$E..S#.F.VYSAKKFPGL..ST..SR.!AEQ

                                              Velvet domain
                          ____________________________________________________________
                          190        200       210       220       230       240
ANIA_01052_AnVeA          GCRVRIRRDVRMRRRGDKRTEDYDYDNERGYNNRRPDQYAGSDAYANAPERPRSTSISTN
Bcin15g03390_BcVel1       GCRVRIRRDVRMRRRDTKPGGDFGEKEDE.YQQGRATS..PPFDYNIQAARQRALSASVH
sscle_11g084630_SsVel1    GCRVRIRRDVRMRRRDTKPGGDFGENEDE.YQQGRATS..PTFDYAIQAARQRALSTSGN
consensus>70              GCRVRIRRDVRMRRR..K...D%...##..Y##.R.........Y.....R.R..S.S..

                          250        260       270       280       290       300
ANIA_01052_AnVeA          MDPYSYPSRRPSAVEYGQPIAQPYQRPMASTPAPS....STPIPAPIPMPGPVALPPSTP
Bcin15g03390_BcVel1       EDPQQR.........RGSGEISPYHSPVVNTPFRTPSISPSTPNAPLPPGNQLGWIPNGP
sscle_11g084630_SsVel1    EDPQQR.........RGSGEISPYRSPAAGSPYRTPSLSPSTSNAPLPAGGQLHWMAQGS
consensus>70              .DP............G.....PY..P....P............AP.P...........

                              310        320       330       340       350       360
ANIA_01052_AnVeA          SPASAHAPAPPSVPLAAPPPLHTPSYQSHLSFGATQTQYPAPQLSHIPQQTTTPTHPYSP
Bcin15g03390_BcVel1       GYAAAPSIQPPHPPPP.....PPSSYPQSMPATH.HNQGPSTQFRQQPPQGPPPA...PI
sscle_11g084630_SsVel1    GYAPVQAIQPPPPPPPPPP..PSSSYSQPISVAY.PNQGPSTPFRQQALQGPPPP...PP
consensus>70              ..A......PP..P..........SY...........Q.P.........Q...P......

                              370        380                           390
ANIA_01052_AnVeA          RSSISHSRNQSISEYEPS.................MGYP......GSQTR.LSAERPSYG
Bcin15g03390_BcVel1       GY...DERRSSYSQFRPPTNPSQQQSYESDYRRMSFGYQIPASSQGPQPIAPAVQNPAYN
sscle_11g084630_SsVel1    GSYSGFEERQSFSQFRPPANPPQPQSYDPDYRRMSFAYQMSNASQGSQPTTPAVQNSTYS
consensus>70              .........S.S#%.P.....................Y......G.Q......#...Y.

                        400                 410        420       430       440
ANIA_01052_AnVeA          QPS.................QTTSLPPLRHSLEPSVNSRSKTPSNMITSLPPIQSLSEL
Bcin15g03390_BcVel1       QQSMEPTYSRNPP.AYSTSFQDSVALAPLRAAEQPLAMS..........PLASVTSISRG
sscle_11g084630_SsVel1    QQSMEPTYSRNPSSAYSNSYQDSVALAPLRA.EQPTAMS..........PLAPVTSMIRG
consensus>70              Q.S..................#...L.PLR...#P...S...........L..!.S....

                                450                 460       470       480       490
ANIA_01052_AnVeA          PSTTSQPSSAIGSSP..........ANEPGPRLWETNSMLSKRTYEESFGHDDRPLYNGM
Bcin15g03390_BcVel1       TQ.NSAPMPS....HNYNKLERSGSYSQYAPIEAEAPKSTNKRSFNDVFSTPTESLSNGR
sscle_11g084630_SsVel1    PP.SLAPMPSIGLSNNYNKLERSGSYSQYPPIEAEPPRSANKRSYSDVFITPSESLYNGR
consensus>70              ......P...................#..P...E......KR.%.#.F......L.NG.

                                500       510       520       530
ANIA_01052_AnVeA          RPDSESYPGGMQRRPSYERSSLLDGPDQMAYKRANGRMVSKPATMR.
Bcin15g03390_BcVel1       RPSAIGIDI........E..ENTRKQEQMIYRRANGNIQNKPAPGLN
sscle_11g084630_SsVel1    RPSAISQRE........EDEEEQRFRDQMTYRRANGSFQRKPAPNLN
consensus>70              RP...............E.........#QM.Y.RANG....KPA....
```

Supplemental Figure S3. Protein sequence alignment of SsVel1 orthologues. Consensus sequences are displayed. Full length amino acid sequences are aligned by Clustal Omega. Alignment is presented by ESPript 3.0.

Supplemental Table S1: List of primers used in this study.

Primer code	Sequence	Usage
SsLAE1-1F	TGGACTCTCACTTGGCAGAA	For generate mutants and genotyping
SsLAE1-2R	CGACTCTAGAGGATCCCGGCCATGGTATAAGCGTCCATTC	
SsLAE1-3F	GTTGGTGTCGATGTCAGCTCTTTGCTCGAGGAAGATTCGT	
SsLAE1-4R	GCAGAGGGGAATCATCAAGA	
SsLAE1-5F	TTTGGATTTGGGGACAGGTA	
SsLAE1-6R	AGCATCATCCCAGTTTCTGC	
SsLAE1-7F	AACCTTGCAATTGCTGCATA	
SsLAE1-8R	ACCTAGGGAGGGAGGAGTGA	
SsVEL1-1F	TCGTGCGAACTACTGGAATG	
SsVEL1-2R	CGACTCTAGAGGATCCCGGTATTTCGGGAATGCTTTTGG	
SsVEL1-3F	GTTGGTGTCGATGTCAGCTCCCCATGCCAAGTATTGGATTA	
SsVEL1-4R	CGCTTAGTAGGAAACTTTAAACTGG	
SsVEL1-5F	CCAGTGTCTGGAATGGCATA	
SsVEL1-6R	GGAGAAAGCGAAGGTGTACG	
SsVEL1-7F	TAGGTCGCATTGCAGAGAAA	
SsVEL1-8R	AACAAACCGAGAAACCGTTG	
H855-nF	GACGGCAATTTCGATGATG	Primers specific for *hph* gene, used for genotyping
H855-nR	CTGCTACAAGTGGGGCTGAT	
